# BNIP3 as a potential biomarker for the identification of prognosis and diagnosis in solid tumours

**DOI:** 10.1186/s12943-023-01808-9

**Published:** 2023-08-30

**Authors:** Qin Yu, Wenhao Fu, Yutang Fu, Wenjing Ye, Huiqiong Yan, Zecheng Yu, Ruirui Li, Yili Cai, Yuxin Chen, Lingyun Wang, Xianqiao Wei, Yangkun Chen, Yuheng Zhang, Huazhong Ying, Furong Tang, Fangwei Dai, Wei Han

**Affiliations:** 1 School of Information Engineering, Hangzhou Medical College, Hangzhou, Zhejiang China; 2 School of Medical Imaging, Hangzhou Medical College, Hangzhou, Zhejiang China; 3 Center of Laboratory Animal, Hangzhou Medical College, Hangzhou, 310013 Zhejiang China; 4 Zhejiang Provincial Key Laboratory of Laboratory Animals and Safety Research, Hangzhou Medical College, Hangzhou, 310013 Zhejiang China; 5 School of Clinical Medicine, Hangzhou Medical College, Hangzhou, Zhejiang China; 6 School of Medical Laboratory and Biological Engineering, Hangzhou Medical College, Hangzhou, Zhejiang China; 7 Engineering Research Center of Novel Vaccine of Zhejiang Province, Hangzhou Medical College, Hangzhou, 310013 Zhejiang China; 8 The Quzhou Affiliated Hospital of Wenzhou Medical University, Quzhou People’s Hospital, Quzhou, Zhejiang China; 9 Department of Basic Medical Sciences, School of Medicine, Tsinghua University, Beijing, China

**Keywords:** BNIP3, Immune infiltration, Pan-cancer analysis, Prognostic biomarker, Diagnosis

## Abstract

**Background:**

Traditional radiotherapy and chemotherapy have been intensively studied for their role in the treatment of tumours. However, these therapies often cause side effects for patients, which calls for the development of novel treatment options for tumours. B-cell lymphoma-2 (Bcl-2)/adenovirus E1B 19 kDa-interacting protein 3 (BNIP3) reportedly apoptosis-inducing effects in tumour cells and is associated with the progression and treatment of multiple tumours. Nevertheless, little is known about its potential role in tumour diagnosis and targeted therapy.

**Findings:**

The results of the study demonstrated that the interaction of BNIP3 with HDAC1 may affect the progression of breast invasive cancer (BRCA), sarcoma (SARC), kidney renal clear cell carcinoma (KIRC), and low-grade glioma (LGG). BNIP3 seemed to exert its effects in BRCA and SARC primarily through gene silencing and integrator complex, and in KIRC and LGG, mainly by affecting olfactory function, suggesting that targeted therapy can be developed based on the above signalling pathway and downstream molecules.

**Interpretation:**

BNIP3 has emerged as a promising therapeutic and diagnostic target for BRCA, SARC, KIRC, and LGG, providing new insights into tumour molecular therapies in the clinic.

**Supplementary Information:**

The online version contains supplementary material available at 10.1186/s12943-023-01808-9.

As a malignant disease, cancer is a serious global public health issue that has permanently affected human society. Cancer remains the leading cause of death in China despite the improvement in health care and increases in funding for cancer control over recent years [1]. Unfortunately, the immune system of cancer patients is generally significantly compromised and altered after long and painful conventional treatments, such as chemotherapy and radiotherapy [2]. As a result, attention is shifting to emerging immune cell therapies because of their advantages of targeting, high efficiency, gene editing, and few side effects. The current study analysed the potential of -cell lymphoma-2 (Bcl-2)/adenovirus E1B 19 kDa-interacting protein 3 (BNIP3) as a potential prognostic marker for tumours.

BNIP3 is a mitochondrial pro-apoptotic protein in the Bcl-2 superfamily, which is vital for the induction of tumour cell apoptosis, as well as the regulation of mitochondrial autophagy, apoptosis, and other functions in tumour cells. Of note, Rossi et al. demonstrated that BNIP3 modulated melanoma development through gene silencing and autophagy [3]. Gorbunova et al. also observed that BNIP3 promoted tumour growth in lung cancer by reducing cell autophagy [4]. Common treatments for cancers, particularly breast invasive cancer (BRCA), sarcoma (SARC), kidney renal clear cell carcinoma (KIRC), and low-grade glioma (LGG), include surgical resection, radiotherapy, chemotherapy, targeted therapy, and immunotherapy. Studies have shown that BNIP3 can affect the therapeutic outcomes of radiotherapy and chemotherapy, as well as serve as a biological marker for targeted therapy, in the treatment research for these four cancers [5]. Nonetheless, the molecular mechanisms of BNIP3 in tumour therapies are poorly understood.

Accordingly, the present study used a multi-omics database and R language to conduct differential, survival, clinical correlation, microsatellite instability (MSI), and correlation analyses of BNIP3 expression in 33 pan-cancerous tumours, explicating the tumour mutational burden (TMB), tumour microenvironment, and immune cell infiltration. The results showed that BNIP3 expression in tumour samples was markedly lower than that in normal samples and that the low expression of BNIP3 was associated with the poor prognosis of BRCA, SARC, KIRC, and LGG. To investigate the molecular mechanism of BNIP3 in these tumours, the signalling pathways of BNIP3 were further analysed through protein–protein interaction (PPI) and gene set enrichment analyses (GSEA). Subsequently, we explored treatments that target BNIP3 by studying the molecules involved in its signalling pathways to provide new ideas for tumour diagnosis and treatment.

## Results and discussion

**Fig. 1:**
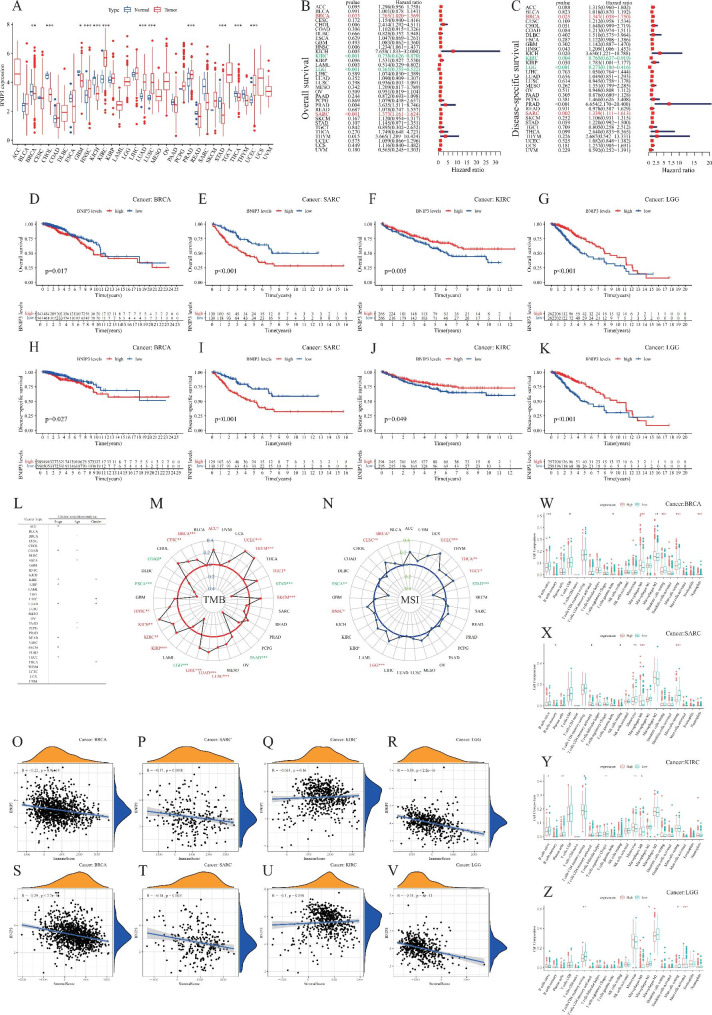
BNIP3 expressed prognostic clinical tumour biology in pan-cancer, and immune microenvironments in BRCA, SARC, KIRC, and LGG. **A**: BNIP3 mRNA expression in different types of cancer. **B–C**: COX regression analysis. **D–K**: Kaplan–Meier survival analysis. **L**: The relationship between BNIP3 expression and three clinical characteristics (age, gender, and stage) in 33 types of cancers. **M–N**: Correlation between BNIP3 and MSI. **O–Z**: The relationship between BNIP3 expression and different types of immune cell infiltration levels in cancers

Transcriptomic, mutation, and clinical cancer data were downloaded from the UCSC Xena (http://xena.ucsc.edu/) database containing 10,327 cancer samples and 730 normal samples, which yielded 11,057 transcriptomic data. We used the R language to perform gene ID conversion on the downloaded gene data, extract the BNIP3 gene from the transcriptome data, and form tables of sample names, gene expression, sample types, and tumour types. The data were also analysed in series using R packages limma, ggpubr, etc. There were no available samples for some cancer types. Among these 33 cancers, 23 exhibited low BNIP3 expression, whereas 10 had extremely significantly high expression of BNIP3 (Fig. 1A), indicating that the abnormal expression of BNIP3 may be involved in the development of multiple malignancies. In the prognostic analysis with the forest plot (Fig. 1B, C), low BNIP3 expression was correlated with overall analysis (OS) in 10 tumours and disease-specific survival (DSS) in 7 tumours, illustrating the prognostic significance of BNIP3.

The clinical data of individual cancers were utilised for analysis, and the results of Kaplan–Meier survival curves (Fig. 1D-K) exhibited that poor prognosis was associated with BINP3 upregulation in patients with BRCA (P = 0.017) and SARC (P < 0.001) and with BNIP3 downregulation in patients with KIRC (P = 0.005) or LGG (P < 0.001). BNIP3 expression varied by stage, age, and sex (Fig. 1L). As previously reported, more pronounced TMB and MSI suggest that tumour cells produce more neoantigens that can be recognised by immune cells as foreign substances, thus inducing anti-tumour immune responses [6]. In BRCA and KIRC, BNIP3 expression was significantly positively correlated with TMB and MSI, that is, patients with higher BNIP3 expression in this tumour also had higher TMB and longer OS. By contrast, BNIP3 expression was negatively and positively correlated with TMB and MSI, respectively, in LGG.

A prior study reported that TMB was correlated with DNA damage/repair pathways and that higher TMB levels represented higher levels of DNA damage [7]. Therefore, correlation analyses of BNIP3 expression with tumour microenvironment and immune cell infiltration were conducted. In the correlation analysis of the tumour microenvironment, the correlation of BNIP3 expression with immune and stromal cell levels was positive in BRCA (r = -0.22, r = -0.29), SARC (r = -0.17, r = -0.18), LGG (r = -0.39, r = -0.31) but negative in KIRC (r = -0.061, r = -0.1) (Fig. 1O–V), implicating that BNIP3 upregulation was associated with lower immune and stromal cell levels and higher tumour cell purity, which could lead to shorter survival of patients [8]. Regarding immune cell infiltration, BNIP3 expression was substantially correlated with immune cells, especially mast cells, in BRCA (r = -0.142, P < 0.001), SARC (r = -0.27, P < 0.001), and LGG (r = -0.166, P = 0.004). Mast cells, innate immune cells in tissues, are important for tissue homeostasis and inflammation. Mast cell proliferation shares an association with certain pathological conditions, and mast cells are associated with several health and disease states [9]. Our analysis also confirmed that BNIP3 expression modulated mast cells and the tumour microenvironment, thus affecting patient survival.

**Fig. 2:**
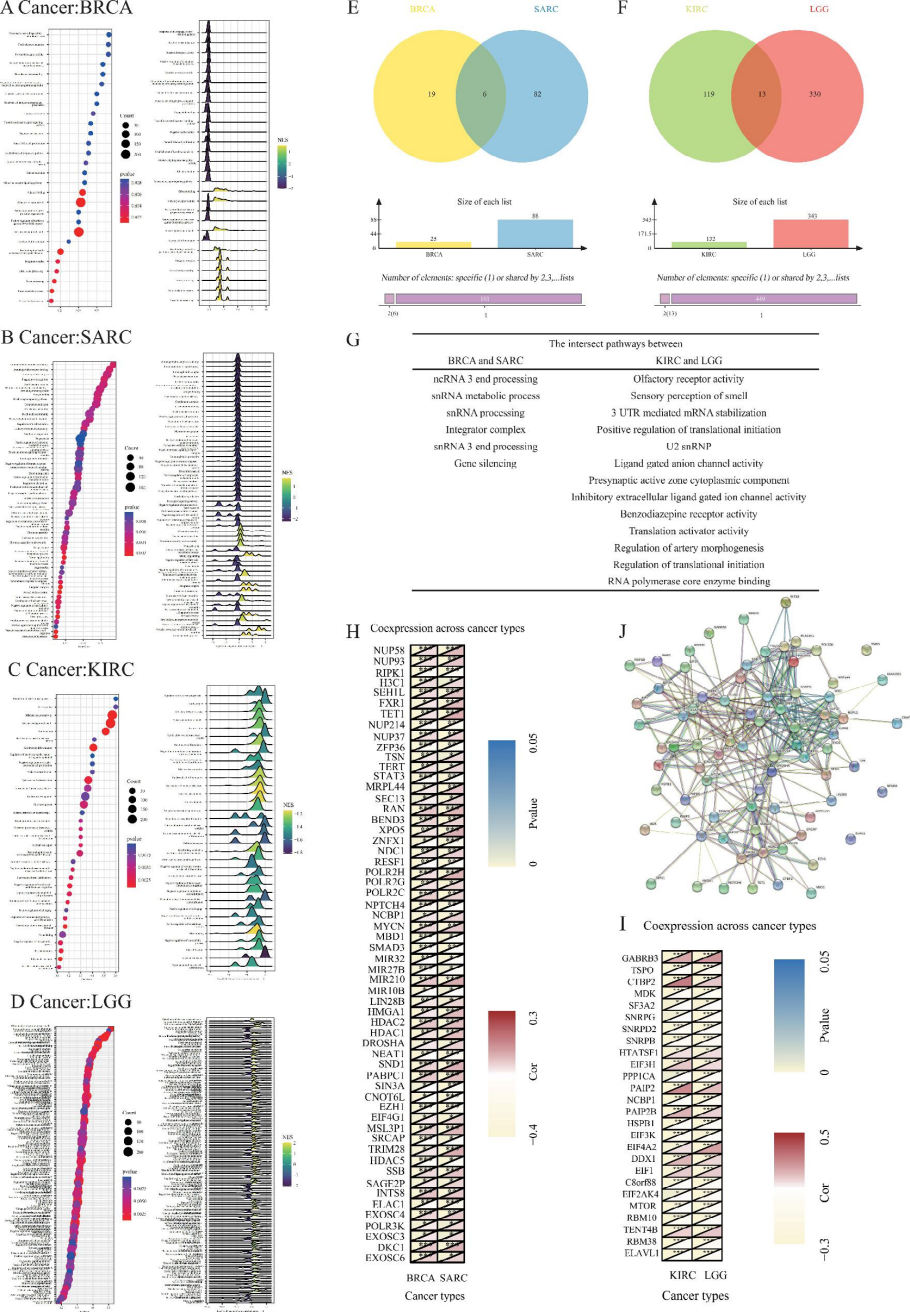
GSEA and Venn diagram and PPI gene co-expression mining BNIP3 as a diagnostic molecular marker for BRCA, SARC, KIRC, and LGG. **A**: Signalling pathways associated with BNIP3 expression in BRCA. **B**: Signalling pathways associated with BNIP3 expression in SARC. **C**: Signalling pathways associated with BNIP3 expression in KIRC. **D**: Signalling pathways associated with BNIP3 expression in LGG. **E**: Venn diagram of KIRC and LGG. **F**: Venn diagram of KIRC and LGG. **G**: The intersect pathways between SARC and BRCA and between KIRC and LGG. **H-I**: Correlation between BNIP3 expression and related gene (immune checkpoint genes and interacted genes) expression. **J**: PPI network for BNIP3-interacting genes

BNIP3 exerts its effects on the occurrence of different types of cancer through various biological processes. We explored potential pathways and genes through which BNIP3 may affect tumourigenesis of the four tumours using the GSEA and Venn diagram analysis. The results showed that BNIP3 regulated the tumourigenesis of BRCA and SARC primarily through six pathways, including ncRNA 3′ end processing, snRNA processing, integrator complex, snRNA 3′ end processing, snRNA metabolic process, and gene silencing (Fig. 2A–G). Further, BNIP3 affects the tumorigenesis of KIRC and LGG mainly via UTR mediated mRNA, positive regulation of translational initiation, regulation of translational initiation, U2 snRNP, regulation of artery morphogenesis, presynaptic active zone cytoplasmic component, inhibitory extracellular ligand-gated ion channel activity, translation activator activity, benzodiazepine receptor activity, and ligand-gated anion channel activity pathways.

PPI analyses were further conducted to probe the molecular mechanism of action of BNIP3 in tumours. The PPI network revealed that an interaction existed between BNIP3 and histone deacetylases (HDACs), which are involved in several core processes regulating endothelial cell physiological functions, especially angiogenesis, inflammatory signalling, and redox homeostasis (Fig. 2H–I). Most endothelial HDACs have anti-proliferative functions, among which class I HDACs promote the proliferation of cancer cells. In addition to different external stimuli, external stressors also affect whether HDAC1 functions in pro- or anti-inflammatory pathways [10, 11]. This fact also explains, to some extent, the phenomenon that the high expression of BNIP3 in different tumours plays different roles in tumour metastasis [12], which is one of the major causes of death in cancer patients. Therefore, we hypothesised that BNIP3 also interacts with HDACs to contribute to cancer cell proliferation and metastasis.

In KIRC and LGG, the prognosis of patients with low BNIP3 expression was poorer than that of patients with high expression (Fig. 1B–C), and BNIP3 expression varied between men and women in different subgroups of KIRC (Fig. 1L). HDACs are responsible for neuronal survival [13], and BNIP3 can maintain intra-mitochondrial homeostasis by degrading damaged mitochondria through mitochondrial phagocytosis and is implicated in renal cell survival and renal function stability [14, 15]. How BNIP3 affects the olfactory pathway, leading to changes in olfactory function, will be further explored in future studies as a diagnostic basis for the aforementioned four types of cancer.

Alterations in olfactory function can be affected by various factors, including environmental factors and variations. Therefore, biological correlation studies did not imply any causal relationship. Meanwhile, as a necessary condition for developing a detection method in tumour and eliminating inequality in cancer treatment, this study needs to encompass a broader population and include a wider range of cancer types, including less prevalent ones. Future validation of BNIP3 in large sample sizes is warranted prior to its clinical application as a diagnostic biomarker.

## In conclusion

BNIP3 exhibited abnormal expression in 33 tumours, which is associated with poor prognosis in BRCA, SARC, KIRC, and LGG. Clinical tumour prognosis evaluation through the exprssion of BNIP3 in BRCA, SARC, KIRC, and LGG revealed a potential for favourable immunotherapeutic efficacy of BNIP3 in these tumours. Furthermore, a correlation analysis was conducted on immune microenvironment indicators. Multi-omics GSEA and other approaches indicateds that BNIP3 interacted with HDAC1 and affected tumour prognosis by influencing olfactory function, suggesting that targeted regulation of olfactory function by BNIP3 and HDAC1 may serve as a potential clinical therapy prognostic diagnosis in BRCA, SARC, KIRC, and LGG.

### Electronic supplementary material

Below is the link to the electronic supplementary material.

Supplementary Material 1: **Fig. 1**: Among the four types of tumours (BRCA, SARC, KIRC, and LGG), the correlation between the three clinical features and BNIP3 expression was significant.

Supplementary Material 2: **Table 1**: Total data of differential expression analysis and survival analysis of BNIP3 in 33 types of tumours.

Supplementary Material 3: **Table 2:** The full names and abbreviations of 33 types of tumours correspond to the table.

## Data Availability

The data supporting this study’s findings are openly available in Xena Ucsc at http://xena.ucsc.edu/. The data supporting this study’s findings are openly available in Bioconductor at http://master.bioconductor.org/. The data supporting this study’s findings are openly available in String at http://cn.string-db.org/. The data supporting this study’s findings are openly available in Evenn at http://www.ehbio.com/test/venn/#/.
