# Supplementary figures and images for: BNIP3 as a potential biomarker for the identification of prognosis and diagnosis in solid tumours
Source: Mol Cancer. 2023 Aug 30;22:143. doi: 10.1186/s12943-023-01808-9 (PMC10466744; doi:10.1186/s12943-023-01808-9)

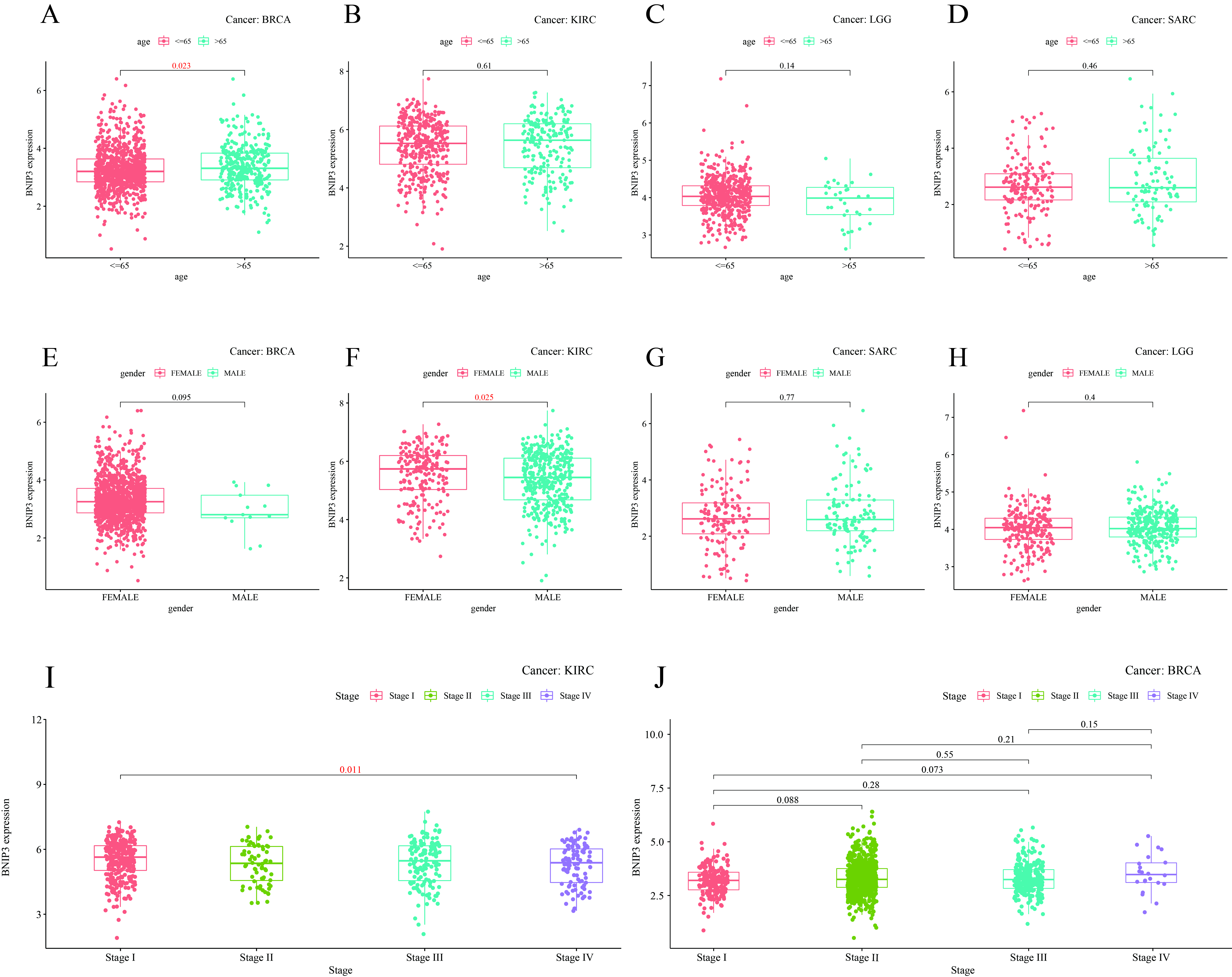

Supplement: Supplementary file 1 — Supplementary Material 1: Fig. 1: Among the four types of tumours (BRCA, SARC, KIRC, and LGG), the correlation between the three clinical features and BNIP3 expression was significant. [file 12943_2023_1808_MOESM1_ESM.jpg]

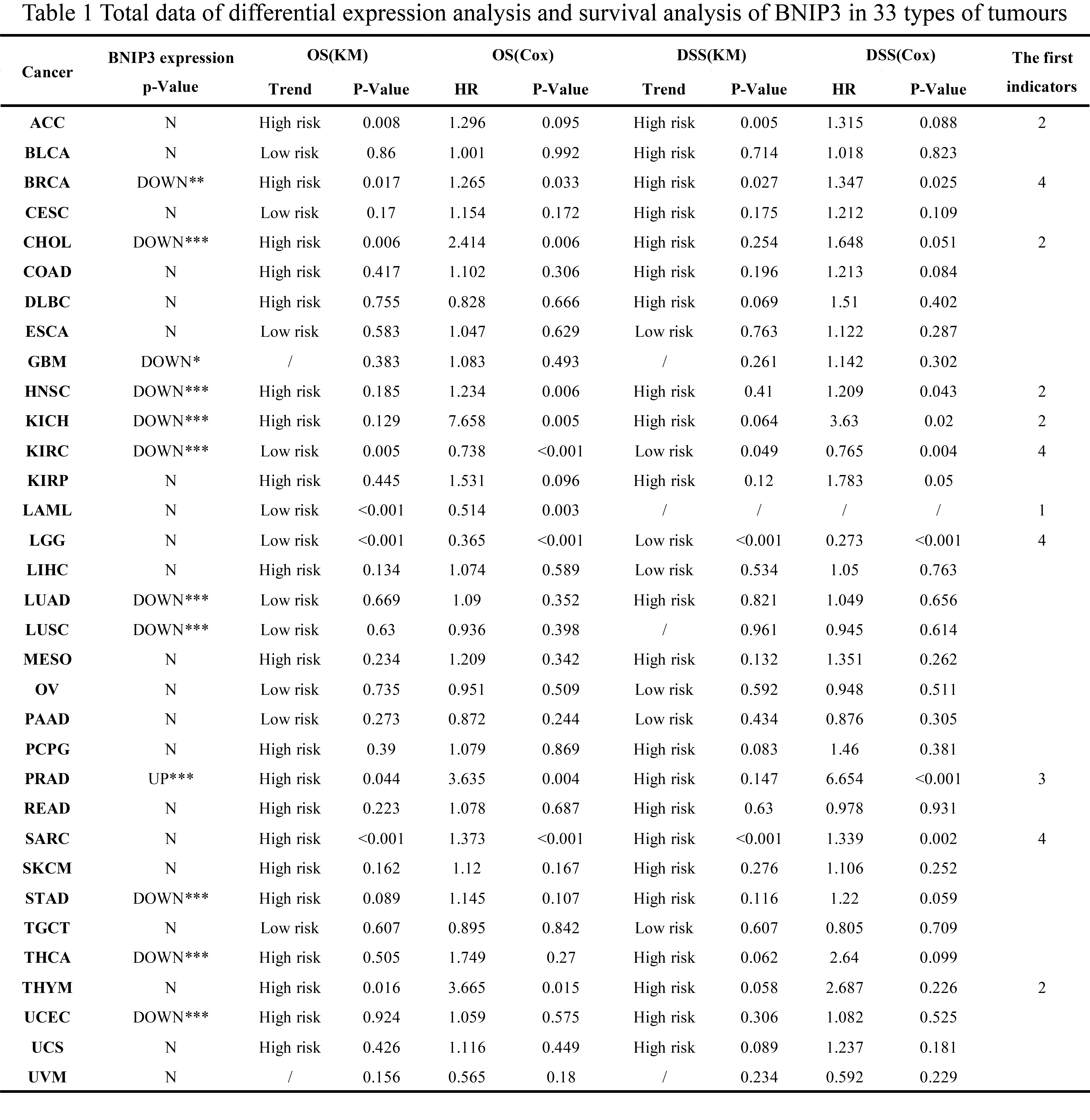

Supplement: Supplementary file 2 — Supplementary Material 2: Table 1: Total data of differential expression analysis and survival analysis of BNIP3 in 33 types of tumours. [file 12943_2023_1808_MOESM2_ESM.jpg]

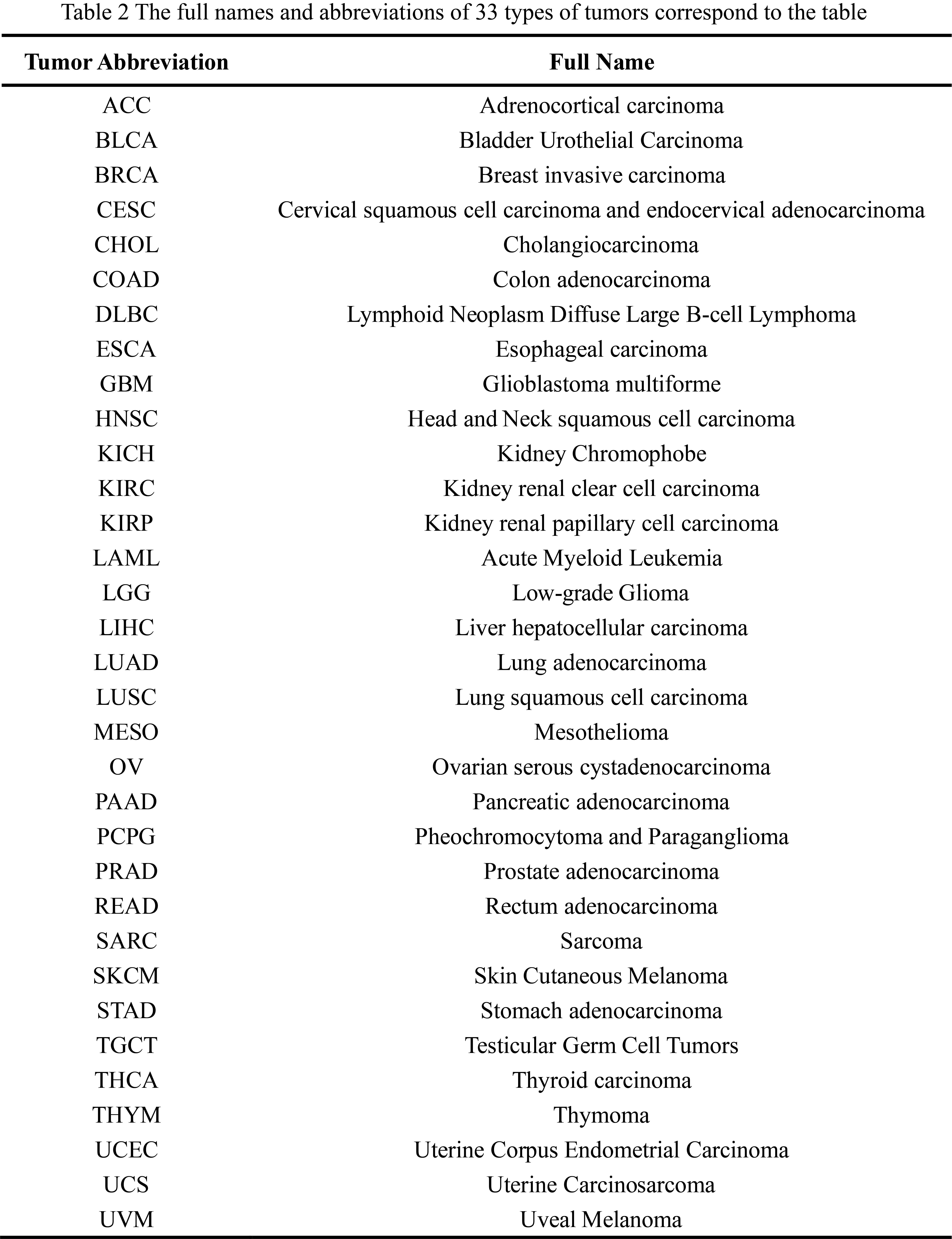

Supplement: Supplementary file 3 — Supplementary Material 3: Table 2: The full names and abbreviations of 33 types of tumours correspond to the table. [file 12943_2023_1808_MOESM3_ESM.jpg]
